# Clinical and Functional Predictors of Response to a Comprehensive Pulmonary Rehabilitation in Severe Post-COVID-19 Patients

**DOI:** 10.3390/microorganisms9122452

**Published:** 2021-11-28

**Authors:** Marc Spielmanns, Melissa Masha Buelow, Anna Maria Pekacka-Egli, Mikis Cecon, Sabine Spielmanns, Wolfram Windisch, Matthias Hermann

**Affiliations:** 1 Pulmonary Medicine and Sleep Medicine Center, Zurich RehaCenter Klinik Wald, CH-8636 Wald, Switzerland; melissa.buelow@gmx.de (M.M.B.); annamaria.pekacka@zhreha.ch (A.M.P.-E.); mikis.cecon@zhreha.ch (M.C.); sabine.spielmanns@zhreha.ch (S.S.); 2 Department of Pulmonary Medicine, Faculty of Health, University Witten-Herdecke, D-58455 Witten, Germany; windischw@kliniken-koeln.de; 3 Neurological Rehabilitation, Zurich RehaCenter Klinik Wald, CH-8636 Wald, Switzerland; 4 Department of Pneumology, Cologne Merheim Hospital, Kliniken der Stadt Koeln GmbH, D-51109 Koeln, Germany; 5 Department of Cardiology, University Heart Centre, University Hospital Zurich, CH-8006 Zurich, Switzerland; matthias.hermann@usz.ch

**Keywords:** COVID-19, pulmonary rehabilitation, FIM, 6-MWD, FVC

## Abstract

Background: Pulmonary rehabilitation (PR) following severe and very severe COVID-19 infection is known to be effective, according to typical assessments. However, not all patients benefit from PR to the same extent. This analysis aimed to identify the impact of different factors on PR outcomes in post-COVID-19 patients. Methods: This prospective observational study included 184 post-COVID-19 patients. The achievement of the predicted reference walking distance (6 min walking distance (6-MWD)) served as a parameter with which to identify responders and non-responders to PR. Several parameters (e.g., Functional Independent Measurement (FIM); pulmonary function testing (Forced Vital Capacity, FVC); 6MWD) were assessed in order to estimate their impact on PR success. Logistic regression models and classification and regression trees were used for multivariate analysis. Results: A total of 94 patients (51%) reached their reference 6MWD by the end of PR. FVC (0.95 (0.93–0.97)), 6MWD at admission (0.99 (0.99–1.00)), and FIM motoric (0.96 (0.93–0.99)) correlated with the risk not reaching the reference distance. The most important variable was the 6MWD at admission. Classification and regression tree identified 6MWD ≥ 130 m at admission and FVC predicted of >83% as the strongest predictor for reaching predicted 6-MWD. Conclusion: Post-COVID-19 patients with lower 6MWD, lower motoric FIM scores and lower FVC at admission have a high risk of not reaching their target values of physical performance despite intensive rehabilitation. As well as identifying them, it is of utmost importance to develop optimal PR concepts for these patients.

## 1. Introduction

Pulmonary rehabilitation (PR), as a comprehensive intervention of exercise training, education, and behavior change, has positive effects on the progress of various pulmonary diseases, particularly for patients with chronic obstructive pulmonary disease (COPD) [1,2]. Even in seriously ill patients awaiting lung transplantation, significant and clinically relevant improvements can be achieved by PR [3].

Comprehensive pulmonary rehabilitation (PR) treatment in the sub-acute phase of COVID-19 ensures continuity of care and results in good recovery in a short time and a reduction in the length of stay on acute wards [4]. PR has already been reported to be safe, feasible and effective [5]. A recent study demonstrates that improvements during PR were significantly higher for post-COVID-19 patients in comparison to patients with other lung diseases usually referred to PR [6], which was also shown for post-intensive-care unit patients suffering from COVID-19 [7]. This was not only true for the physical performance measured with Functional Independence Measurement (FIM) and 6 min walking distance (6-MWD), but also for the wellbeing of the patients, as indicated by the results of the Feeling Thermometer (FT).

It is already known that COVID-19 infection is not only a respiratory disease, as it can affect different body systems and functions, causing severe and complex disability, especially in more severe patients with prolonged hospitalization in ICUs or acute medical wards. The functional limitations of severe post-COVID-19 patients usually require an inpatient PR setting. Few publications suggest the provision of a comprehensive outpatient PR as an alternative setting for the anticipated respiratory impairments, e.g., muscle atrophy associated with physical deconditioning, as well as other negative consequences of COVID-19, including fatigue and hospital-induced anxiety or depression [8].

It is worthy of note that not all patients with pulmonary disease benefit from PR to the same degree. In COPD patients, the response rate was approximately two thirds [9,10] and the non-responder rate in patients with interstitial lung diseases (ILD) was approximately 40%. Both COPD and ILD patients with major limitations in the 6MWD at the beginning of the PR benefited the most from PR [10].

To date, there are no studies available on the response rates of post-COVID-19 patients to PR. Although there are some data showing the impressive benefits of a PR participation, the question of why some post-COVID-19 patients do not improve sufficiently despite participation remains unanswered. Therefore, we performed this study to identify patients who do not improve sufficiently and to find potential reasons for this.

## 2. Materials and Methods

### 2.1. Participants and Procedures

Patients overcoming the acute phase of COVID-19 infection were referred from acute hospitals for PR to the Zurich RehaCenter, Klinik Wald, Switzerland after hospitalization between March 2020 and May 2021. Post-COVID-19 patients were eligible for PR as soon as they were hemodynamically stable without the need of catecholamine or invasive ventilation and continuous monitoring. The data of these patients were prospectively analyzed according to performance and outcome during rehabilitation. We used the German version of the program RehaTIS^TM^ by Softsolution, International AG, 15830 Lahti, Finland to record and control the individual rehabilitation process of each participant, including all therapies and procedures. The patient data and the results of the assessments were stored and taken for evaluation out of the clinic information system Phoenix^TM^, CompuGroup Medical AG, 3007 Bern, Switzerland.

The patients were defined as responders if they achieved a walking distance (6-MWD) in the 6 Minute Walking Test (6-MWT) that corresponded to the reference walking distance by participating in the rehabilitation program [11]. The resulting gender-specific regression equations were used for men,

6MWD = (7.57 × height cm) − (5.02 × age) − (1.76 × weight kg) − 309 m,

and for women,

6MWD = (2.11 × height cm) − (2.29 × weight kg) − (5.78 × age) + 667 m.

All the patients who evaluated provided written informed consent, and the local ethics committee approved the study protocol (BASEC-No 2020-01061). The data from patients who did not give their consent were not included in this analysis. This study was registered at the German Clinical Trials Register (DRKS00024613).

The patients referred to PR were assessed with questionnaires, such as the Chronic Respiratory Disease Questionnaire (CRQ), the Hospital Anxiety and Depression Scale (HADS), the Cumulative Illness Rating scale (CIRS), and the Functional Independence Measure (FIM) within 2 days after admission for rehabilitation. In order to evaluate the response to PR, 6MWD, FIM, and Feeling Thermometer (FT) were performed at admission and before discharge. All the patients were deemed cognitively able to provide valid responses to the questionnaires by treating physicians. Comorbidities, pulmonary function testing (PFT), and laboratory values including blood gas analysis were assessed.

### 2.2. Pulmonary Rehabilitation

The usual duration of this inpatient PR program was 3 weeks and consisted primarily of an individualized endurance exercise and strength training. Contents were adapted to the patients functional performance and physical limitations. All over 25–30 therapy sessions on 5–6 weekdays were performed consisting of 4 exercise sessions per day. On Saturday, one exercise session was offered, while on Sunday no exercises were provided.

Details of the PR program have been described previously by our group [5,6]. In brief, exercise therapy included endurance training (cycling and treadmill), gymnastics (3 levels), in- and outdoor walking (3 levels), and strength training.

Partially bedridden patients started with in-bed cycling and MOTO-Med^®^ (RECK-Technik GmbH & Co. KG Medizintechnik, Reckstraße 1–5, D-88422 Betzenweiler, Germany). This was followed by initial walking attempts with walking aids until the first cycling interval training was possible. Walking training outside the clinic was offered at different levels, with level 1 at a slow pace with little incline and level 3 at a faster pace with frequent inclines. Gymnastics were offered at three levels of intensity. Gymnastics at level 1 took place mostly in a sitting position with several breaks between exercises; level 3 consisted of exercises in a standing position or walking with only very few or no breaks at all between exercises. The gymnastics consisted of a mixture of exercises to improve endurance, strength, coordination, range of motion, and balance.

Strength exercises were additionally performed 3–4 times per week individually according to recent American Thoracic Society/European Respiratory Society recommendations [1]. Exercise intensity was also symptom-controlled by Borg Scale (goal of Borg 4–5/10) [12,13].

Twice a week (1 h each), all the patients participated in educational sessions, including self-management, coping skills, self-medication, management of infections and exacerbations, dyspnea, use of oxygen, and nutrition interventions. If needed, the patients took part in a structured smoking cessation program, and received psychosocial support or diabetes advice.

### 2.3. Exercise Capacity

Exercise capacity was measured (6MWD) using the 6MWT, performed once a week, according to the guidelines of the American Thoracic Society and carried out by experienced, well-instructed examiners [14]. Regarding the relationship between the 6MWT distance and health outcomes, the inverse association with adverse outcomes was previously demonstrated in disease-specific populations such as those with heart failure, pulmonary disease, end-stage liver disease [15,16,17,18].

### 2.4. Quality of Life

As the standardized health-related quality of life (HRQoL) measurement tool, the German version of the Chronic Respiratory Disease Questionnaire (CRQ) was used. The 20 items represent areas of dysfunction that are most significant to patients with chronic respiratory diseases. The patients completed the CRQ independently. Four aspects of HRQoL were evaluated: dyspnea, fatigue, emotional function, and mastery. Each domain included 4 to 7 items, graded on a 7 point Likert scale. The item scores within a domain were summated to provide a total score for each domain. While a higher score indicated a better HRQoL, the minimally important difference was reflected by a change in score of 0.5 on a 7 point scale. In previous research, the CRQ was used to evaluate the effects of treatment in clinical trials as well as in clinical practice. Evidence has generally shown that the CRQ is a reliable and reproducible tool [19].

### 2.5. Functional Independence Measure (FIM)

The FIM is an 18 item measurement tool that explores the severity of an individual’s physical and psychological disability, especially in rehabilitation patients [20]. The tool is also used to assess changes in patients’ functional status in response to rehabilitation or medical intervention. The FIM uses the level of assistance for individual needs to grade functional status from total independence to total assistance. As the severity of disability changes during rehabilitation, the data generated by the FIM Instrument can be used to track such changes and analyze the outcomes of rehabilitation. FIM change scores associated with MID were 22 for the total FIM, 17 for motor FIM, and 3 for cognitive FIM, respectively [21].

### 2.6. Hospital Anxiety and Depression Scale (HADS)

The HADS was originally designed as a short, easy-to-use screening tool for depression and anxiety in patient status in response to rehabilitation. This questionnaire is focused on non-physical symptoms and can be used to diagnose depression in people with significant physical limitations. It comprises seven questions for anxiety and seven questions for depression, and takes 2–5 min to complete. Both scales ranging from 0 to 21 with higher scores indicate more severe distress. Cut-off scores are available for quantification, e.g., a score of 8 or more for anxiety has a specificity of 0.78 and a sensitivity of 0.9, and for depression a specificity of 0.79 and a sensitivity of 0.83 [22].

### 2.7. Cumulative Illness Rating Scale (CIRS)

The CIRS is a comprehensive method for recording diseases in 14 organ systems on the basis of an evaluation of 0 to 4 points, with the help of which a cumulative score is calculated. The range of the score is 0–56 points. When evaluating the CIRS, each individual illness in the corresponding organ system must be classified. If there were different diseases within the same organ system, only the disease that was most pronounced was evaluated. The CIRS was used as a morbidity index in order to assess a patient’s level of disability and as an indicator of health status, including predicted 18 month mortality and social function [23]. The calculated CIRS at admission is useful for predicting important hospital outcomes such as high risk of death or long stays and to better anticipate end-of-life issues.

### 2.8. Feeling Thermometer (FT)

We used the FT to determine and compare the patients’ feelings about their wellbeing by applying a numeric rating of their feelings toward an imaginary scale in terms of degrees, with their attitudes corresponding to temperatures. The minimal important difference was defined between 5 and 8 degrees [24].

### 2.9. Pulmonary Function Tests (PFT) and Blood Gas Analysis

Spirometry and Body-Plethysmography (Master Screen Body; Jaeger GmbH, Hoechberg, Germany) were performed once on PR admission, in accordance with recent guidelines [25,26]. Arterial blood gases were taken at rest under room air conditions (Radiometer ABL800, Willich, Germany) on admission to PR [27]. Blood count, creatinine, and C-reactive protein (CRP) were obtained from the external laboratory Medica, Medizinische Laboratorien Dr. F. Kaeppeli AG, Zurich, Switzerland.

### 2.10. Statistics

The continuous variables were represented as mean with standard deviation (SD), and the discrete variables with absolute and relative numbers. The correlations between the continuous variables for pairwise complete observations were calculated using Pearson correlation coefficient. Univariate comparisons between patients who reached the target range and patients who did not reach the target range were performed using t-tests or chi square tests. Logistic regression models and classification and regression trees (CART) were used for multivariate analysis. CART analyses help to divide patients into different groups according to influencing variables. In our case, the division was made into patients who were likely to reach the reference walking distance, those who would not reach the reference walking distance, and groups in between. All the variables with a *p* value < 0.1 and a rate of missing values <25% were selected for multivariate analysis. To avoid multicollinearity variables, variance inflation factors were calculated (VIF). If two variables had a VIF > 4, one variable was selected for modelling. R (Version 4.1.0, Microsoft R Open, Microsoft Corporation, One Microsoft Way, Redmond, WA 98052-6399, USA) was used for all the analyses. A significance level of 5% was set.

## 3. Results

**Figure 1:**
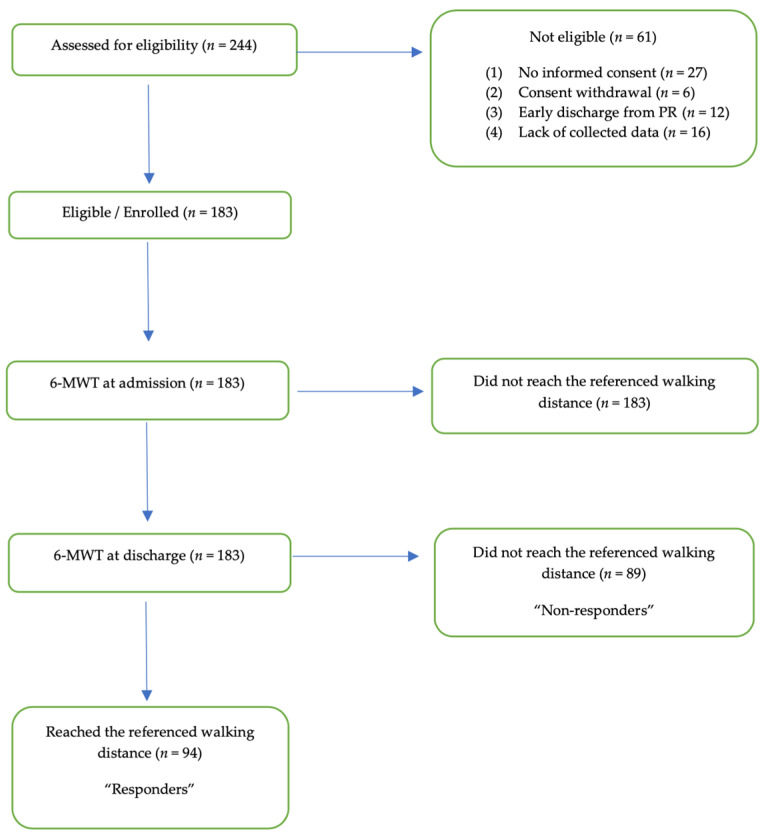
Study design.

Between January 2020 and May 2021, a total of 244 post-COVID-19 patients were referred to PR. In total, 61 patients were excluded from the analysis due to several reasons (*n* = 27 did not give informed consent; *n* = 6 withdraw their consent; *n* = 12 early discharge from PR; *n* = 16 lack of collected data). The remaining 183 patients were evaluated in the study. None of the participants reached the reference walking distance on admission to PR. According to the results of the 6MWT, in the pre–post comparison of all the post-COVID-19 patients, 94 out of 183 patients reached the referencing walking distance at discharge. Those patients were defined as “responders”, whereas the 89 patients did not reach the referenced walking distance, defined as “non-responders”. The chart review process is depicted in Figure 1.

### 3.1. Baseline Characteristics

The overall collective had a mean age of 69 years on average. A total of 32.6% of the patients were female (*n* = 60). According to age and sex, there were no significant differences between responders and non-responders. Acute Hospital stay was 23.5 days on average, with 9.2 days on ICU and 5.7 days on ventilation (Table 1). Oxygen supply at discharge from the acute hospital was needed in 85% of the patients. According to the World Health Organization (WHO) criteria 55% of the patients suffered from a severe disease (with severe pneumonia) that required oxygen therapy and 45% developed a critical disease with complications, such as respiratory failure, acute respiratory distress syndrome, thromboembolism, sepsis, and/or multiorgan failure [28]. Almost half of the patients (*n* = 88) had an initial 6MWT distance <200 m, which required an individual adaptation of training.

### 3.2. Comorbidities

The CIRS in the overall collective was 12.7 points, on average. A relevant number of patients had cardiovascular risk factors, such as arterial hypertension (54%), smoking (29%), diabetes (28%) or established coronary artery disease (17%). Mean BMI was 27 kg/m^2^ (Table 1),

### 3.3. Assessments on Admission

The mean duration of the pulmonary rehabilitation program was 21.6 days, with 1658 therapy minutes per week on average. The responders had a significantly higher total and motoric FIM score at admission than the non-responders (103.2 and 73.0 in responders vs. 92.9 and 64.2 in non-responders; *p* < 0.001 for both). No significant differences were found for HADS, CRQ, Feeling Thermometer, and laboratory parameters (Table 1 and Table 2). Out of 156 correctly answered HADS questionnaires a HADS-D score ≥ 10 points was found in 19 patients (12%) and a HADS-A score ≥ 10 points was also found in 19 patients (12%), with no significant difference in the distribution for both groups.

- Pulmonary function tests on admission

The FEV1% predicted at admission was significantly higher in the responders than in the non-responders (83.2% vs. 63.1%; *p* < 0.001). The same applied to the FVC% predicted (80% in responders vs. 62% in non-responders; *p* < 0.001). The responders demonstrated a higher diffusion capacity on admission compared to the non-responders (DLCO% predicted 62% in responders vs. 47% in non-responders) (Table 2).

- Functional and subjective changes during PR

Improvements for each group during PR are shown in Table 3. Only Δ6-MWD (distance in meters walked on the 6 min walking test at discharge minus distance walked on admission) was different between the responders and the non-responders (175.80 m (109.13) vs. 131.65 m (87.49)). Regarding the groups according to reaching the MCID of the 6MWD, we found no significant differences (Table 3). Changes in 6MWD, total and motoric FIM and FT during PR were significant for the whole cohort, for both responders and non-responders (Table 4). Changes in 6MWD, total FIM, and FT during PR were significant.

Regression models were run once with and once without the PFT parameters, since PFT was performed in only 75% of the patients on admission. In both models, 6-MWD on admission (OR: 0.99 (0.99–1.00) p<0.001; OR: 0.99 (0.99–1.00), *p* = 0.002) and motor FIM (OR: 0.96 (0.93–0.99), *p* = 0.002; OR: 0.93 (0.88–0.97), *p* = 0.004) were significant. In the model with pulmonary function parameters, FVC was also significant (OR: 0.95 (0.93–0.97) *p* < 0.001) (Table 5).

**Figure 2:**
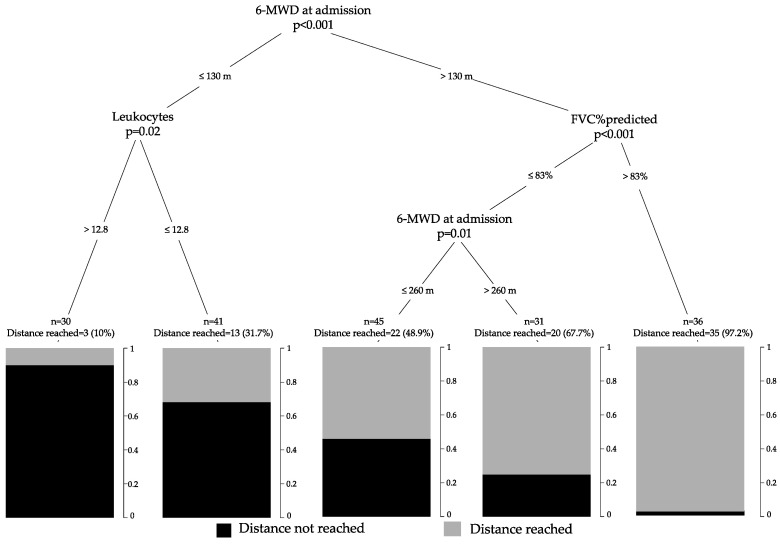
Separation of non-responders into different groups according to influencing variables (tree model). Notes: 6MWD, six-minute walking distance; FVC, forced vital capacity.

The most important variable in the tree model was 6MWD on admission. Here, the split was set at 130 m. The patients with a 6MWD > 130 m on admission and >83% of the reference FVC value were the most likely to reach the predicted walking distance (Figure 2).

## 4. Discussion

Our study demonstrated that patients with severe COVID-19 benefit from rehabilitation to varying degrees. Indeed, patients with lower 6MWD and lower motoric FIM scores on admission combined with a restrictive ventilatory disorder were at increased risk of failing to reach the age- and gender-related target values for 6MWD during a comprehensive PR program.

Interestingly, we did not observe any differences in the baseline characteristics, except for FIM scores and pulmonary function testing parameters. These findings are confirmed by the recent study of Maniscalco et al., which suggested that multidisciplinary rehabilitation may be useful in post-COVID-19 patients regardless of the presence of preexisting cardiorespiratory comorbidities [29]. Several studies emphasize that the presence of comorbidities, complications from an intensive care treatment, and virus-associated pulmonary, cardiac, hematological and neurological damage, challenge the concept of PR [30,31,32]. However, in our study, we did not observe any difference in comorbidities, length of intensive care treatment or laboratory parameters, nor in the intensity of PR, as measured by therapy minutes per week between responders and non-responders.

It is recommended to pay attention to the improvements in 6MWD during PR, which provides an assessment of exercise capacity and can better reflect daily activity than laboratory tests [33]. The 54 m threshold for 6MWD is considered representative of a clinically significant change for patients with chronic respiratory diseases [34]. The majority of our patients (83%) largely exceeded this value at the end of PR, although it has been highlighted that a statistically significant mean increase in 6MWD in a group of study participants is often much less than a clinically significant increase in an individual patient [35]. The fact that a large number of patients who were very limited in their ability to walk upon arrival in our facility were able to reach their individual reference walking distance at the end of the PR program, even in the presence of several comorbidities, reinforces our view that all patients hospitalized for COVID-19 should undergo a multidisciplinary rehabilitation program.

Studies have shown that, usually, the patients with the greatest limitations benefit most from PR [36]. In our study, the patients with the greatest limitations as assessed by 6MWD and FIM were also the patients who did not reach the goal of achieving the set level in walking distance, which is in contrast to the expected results for patients with pulmonary diseases referred to PR so far. We assume that the systemic limitations caused by COVID-19 infection are so pronounced in numerous patients that even a three-week PR with conventional contents is currently not enough to convert patients back to normal. This assumption is supported by the results of Skjorten et al., who recently showed that significant cardiovascular-pulmonary limitations were still detectable while performing ergo-spirometry in 1/3 of the post-COVID patients after 3 months [37]. Additionally, Tudoran C. and Tudoran M. et al. detected alterations in the left ventricular systolic and diastolic function in patients with post-acute COVID-19 syndrome by transthoracic echocardiography, which may also serve as an explanation for the reduction in exercise capacity [38,39].

DLCO%-predicted is supposed to be the strongest independent factor associated with previous severe/critical COVID-19. Compared with non-severe cases, severe patients exhibited a higher incidence of DLCO impairment and experienced greater TLC decrease and 6MWD decline [38]. Our study showed that 6MWD and FVC% predicted were the most important variable to reach the predicted walking distance. In this context, it has been suggested that the severity of pulmonary inflammation may be the reason for impaired PFT in COVID-19 patients [39]. Since there is evidence that exercise training represents a strategy to elicit an anti-inflammation effect, which may help to decrease the risk or progression of several disorders of an inflammatory nature [40], as are the parenchymal and vascular sequelae of COVID-19, we wonder why this subgroup did not benefit the most. One could speculate that the effect should be different because of the systemic inflammation that characterizes the cardiorespiratory diseases [41].

As reported previously, the increase in walking distance in post-COVID-19 patients is significantly higher than in patients who are usually admitted to PR [11,35,42]. At least according to the present results, the cohort of post-COVID patients differed significantly from the patients with lung diseases usually admitted to PR. The limitations in physical capacity according to the results of the initial 6MWD of the post-COVID-19 patients exceed the limitations observed in patients usually participating in PR [6]. Although all the patients benefit from PR on average, we identified a subgroup that benefited less. In our view, it is of particular interest to identify these patients with a high likelihood of lower response in terms of walking distance enhancement early in the onset of PR to pay special attention to these patients. Our tree model analysis might further help to identify these patients at risk. Indeed, a first split was set with a 6MWD at 130m. Patients with a 6MWD ≤130 m and higher leucocytes count (>12.8 × 10^9^/L) demonstrated a high probability of not reaching the individual predicted walking distance, while patients with a 6MWD >130 m and an FVC >83% were most likely to reach the individual predicted walking distance.

The current recommendations of the professional societies for the rehabilitation of post-COVID patients are very similar to the usual recommendations for PR in pulmonary patients. Currently, COVID-19 survivors with pre-existing/ongoing lung function impairment at 6–8 weeks following hospital discharge should receive a comprehensive pulmonary rehabilitation program consistent with established international standards, compared to no pulmonary rehabilitation program [29]. An adaptation of the rehabilitation contents for more limited patients is not planned at present. However, these recommendations only meet the needs of some post-COVID patients. Therefore, further studies should clarify a variation of PR contents or intensify care for post-COVID patients with greater functional limitations. In this context, Carda et al. suggested the provision of PR treatment based on the content that is usually recommended in lung fibrosis, since COVID-19 can also induce a restrictive lung disease [43].

According to the results of our analysis, patients benefit from participation in this PR program not only for the objective parameters according to the performance tests, but also subjectively, according to the results of the feeling thermometer. However, according to our analysis, the credo of “one size fits all” is not applicable to the PR of post-COVID patients. Further studies are needed to develop an individualized PR program, especially in post-COVID patients, to meet individual needs.

## 5. Limitations

This study featured limitations. First, this study represents a cohort of severe post-COVID-19 patients referred to a single rehabilitation center. Therefore, the results could not be transferred to all post-COVID 19 patients or to all PR centers. Second, the results of 24% of the patients admitted to our facilities could not be analyzed, even though a prospective study design was chosen. A more complete coverage of all the patients would have been desirable, but this was not achievable. Third, the study included patients from the first and second wave of the pandemic. At that time, the proportion of patients with comorbidities and older patients predominated. In the current, fourth wave, the composition of patients has changed towards younger and unvaccinated patients, as well as patients with poorer immune response to vaccination. To what extent our results also apply to the typical patients of the fourth wave cannot be estimated. Fourth, the simple consideration of the walking distance from admission to rehabilitation showed limitations. The patients in the non-responder group started PR with 6MWD at 32% of the reference value and were less likely to reach the reference value compared to the responder group starting PR with 6MWD at 73% of reference value. Therefore, the determination of the walking distance alone should not be overinterpreted. However, by adding the PFT values to the results of the initial 6MWD, the predictive value for reaching the reference value increased to 97.2%.

## 6. Conclusions

This study identified a subgroup of COVID-19 patients with lower 6MWD, lower motoric FIM scores, and lower FVC at admission as being at a high risk of not reaching their target values for physical performance, despite intensive inpatient rehabilitation. The identification of these patients allows us to develop optimized concepts within PR for these patients.

## Figures and Tables

**Table 1:** Baseline Characteristics of Responders and Non-Responders.

	Overall	Responder	Non-Responder	*p*-Value
*n*	183	94	89	
Age years (mean (SD))	68.99 (10.21)	69.62 (10.09)	68.45 (10.34)	0.441
BMI kg/m^2^ (mean (SD))	27.25 (5.5)	27.38 (4.5)	27.11 (6.4)	0.746
Sex, female (%)	60 (33)	27 (29)	32 (36)	0.375
ICU-days (mean (SD))	9.23 (11.8)	7.98 (10.6)	10.65 (12.9)	0.129
Ventilation days (mean (SD))	5.65 (9.5)	4.65 (8.0)	6.78 (10.9)	0.132
Hospital days (mean (SD))	23.50 (13.5)	21.46 (11.0)	25.81 (15.4)	0.29
Rehabilitation days (mean (SD))	21.58 (8.7)	21.39 (8.4)	21.80 (9.1)	0.755
Therapy minutes/week	1658 (1268)	1752 (1393)	1563 (1125)	0.316
6-MWD admission % reference value (SD)	53.11 (38.02)	73.06 (35.96)	32.04 (27.36)	<0.001
6-MWD admission meters (mean (SD))	187 (134)	250 (126)	119 (104)	<0.001
Comorbidities				
non-smoker (%)	132 (71.7)	73 (77.7)	58 (65.2)	0.88
COPD *n* (%)	10 (5.5)	3 (3.2)	10 (11.2)	0.3
No COPD	173	91	79	
COPD stage I	1 (0.6)	0 (0.0)	1 (1.2)	
COPD stage II	3 (1.7)	1 (1.1)	2 (2.3)	
COPD stage III	4 (2.2)	1 (1.1)	3 (3.5)	
COPD stage IV	2 (1.1)	1 (1.1)	1 (1.2)	
Alcohol abuse *n* (%)	13 (7.1)	6 (6.4)	7 (7.9)	0.919
Coronary Artery Disease *n* (%)	32 (17.4)	11 (11.7)	21 (23.6)	0.55
Diabetes *n* (%)	51 (27.7)	22 (23.4)	29 (32.6)	0.223
Peripheral Arterial Disease *n* (%)	12 (6.5)	4 (4.3)	8 (9.0)	0.320
Atrial Fibrillation *n* (%)	18 (9.8)	5 (5.3)	13 (14.6)	0.63
Stroke *n* (%)	8 (4.3)	3 (3.2)	5 (5.6)	0.659
VTE *n* (%)	4 (2.2)	1 (0.0)	3 (3.2)	0.453
Dyslipidaemia *n* (%)	34 (18.5)	15 (16.0)	19 (21.3)	0.455
Arterial hypertension *n* (%)	99 (53.8)	47 (50.0)	51 (57.3)	0.400
Pulmonary hypertension *n* (%)	2 (1.1)	1 (1.1)	1 (1.1)	1.000
Psychiatric disease *n* (%)	17 (9.2)	9 (9.6)	8 (9.0)	1.000
Renal failure *n* (%)	31 (16.9)	12 (12.8)	19 (21.6)	0.166
CIRS pts (mean (SD))	12.74 (5.54)	11.76 (5.02)	13.80 (5.91)	0.13
Assessments				
HADS A (mean (SD))	5.28 (3.62)	4.62 (3.07)	5.91 (3.99)	0.30
HADS D (mean (SD))	5.52 (3.24)	5.16 (3.00)	5.88 (3.47)	0.178
CRQ (mean (SD))	4.71 (1.03)	4.70 (1.10)	4.71 (0.97)	0.929
FIM total (mean (SD))	98.12 (15.75)	103.16 (12.33)	92.93 (17.30)	<0.001
FIM socio (mean (SD))	29.70 (5.87)	30.63 (6.44)	28.73 (5.09)	0.29
FIM motoric (mean (SD))	68.68 (12.89)	73.04 (11.05)	64.20 (13.20)	<0.001
FT (mean (SD))	53.79 (16.99)	52.96 (17.05)	54.91 (16.87)	0.468

Notes: ICU, Intensive Care Unit; 6MWD, Six-minute walking distance; CIRS, Cumulative Illness Rating Scale; HADS A, Hospital Anxiety and Depression Scale anxiety; HADS D, Hospital Anxiety and Depression scale depression; FIM, functional independence measurement; CRQ, chronic Respiratory questionnaire; VTE, venous thromboembolism; FT, feeling thermometer; COPD, chronic obstructive pulmonary disease (staging according GOLD guidelines).

**Table 2:** Pulmonary function testing (PFT) and Laboratory Parameters of Responders and Non-Responders.

	Overall	Responder	Non-Responder	*p*-Value
**Pulmonary function testing (PFT)**				
FEV1% pred. (mean (SD))	74.28 (21.59)	83.16 (19.43)	63.07 (18.91)	<0.001
FVC% pred. (mean (SD))	71.88 (20.76)	80.03 (18.60)	61.59 (18.79)	<0.001
FEV1% FVC (mean (SD))	80.28 (10.74)	80.53 (9.24)	79.97 (12.45)	0.760
DLCO% pred. (mean (SD))	56.35 (17.81)	62.33 (17.94)	46.95 (13.02)	<0.001
**Laboratory Parameters**				
PaO2 kPa (mean (SD))	9.55 (6.50)	9.15 (1.81)	9.97 (9.14)	0.433
PaCO2 kPa (mean (SD))	4.53 (0.77)	4.47 (0.72)	4.58 (0.81)	0.405
SpO2 % (mean (SD))	93.46 (2.94)	93.55 (2.80)	93.36 (3.11)	0.658
Procalcitonin ng/mL (mean (SD))	1.44 (4.31)	0.59 (0.74)	2.46 (6.23)	0.25
CRP mg/dL (mean (SD))	139.87 (109.79)	131.78 (97.72)	149.12 (121.76)	0.292
Creatinine mg/L (mean (SD))	118.08 (109.49)	107.92 (78.66)	129.55 (134.53)	0.183
Ferritin mg/L (mean (SD))	1536.85 (1552.31)	1513.36 (1644.27)	1570.07 (1431.46)	0.859
Hemoglobin g/L (mean (SD))	104.47 (23.25)	107.46 (23.88)	101.10 (22.27)	0.65
Leukocytes ×10^9^/L (mean (SD))	12.31 (6.06)	11.53 (5.69)	13.20 (6.34)	0.62
Thrombocytes ×10^9^/L (mean (SD))	211.35 (94.09)	205.67 (93.40)	213.66 (89.01)	0.555
CPK U/L (mean (SD))	415.84 (742.37)	435.63 (762.69)	392.38 (725.81)	0.780
D-dimer µ/L (mean (SD))	5.98 (13.08)	4.67 (8.28)	7.54 (17.09)	0.241
Sodium mEq/L (mean (SD))	136.67 (4.65)	136.53 (4.76)	136.79 (4.57)	0.713
Potassium mEq/L (mean (SD))	4.12 (0.52)	4.02 (0.50)	4.23 (0.51)	0.6
Protein g/dL (mean (SD))	70.74 (47.25)	74.71 (46.68)	65.97 (49.12)	0.605

Notes: CPK, creatien phosphokinase; CRP, C-reactive protein; SpO2, oxygen saturation; DLCO, lung diffusion capacity.FEV1, forced expiratory volume in one second; FVC, forced vital capacity; DLCO, lung diffusion capacity.

**Table 3:** PR Results according to 6-MWD, FT and FIM of responders and non-responders.

	Overall	Responder	Non-Responder	*p*-Value
*n*	183	94	89	
6MWD at discharge % reference value (SD)	98.55 (37.78)	126.87 (22.54)	68.63 (25.56)	<0.001
Δ6MWD meter (mean (SD))	154.20 (101.11)	175.80 (109.13)	131.65 (87.49)	0.003
∆6-MWD meter >54 m (minimal important difference) *n* (%)	156 (84.8)	81 (86.2)	74 (83.1)	0.717
ΔFIM tot (mean (SD))	15.36 (13.99)	14.85 (14.69)	15.76 (13.24)	0.666
ΔFIM motoric (mean (SD))	12.27 (10.79)	10.68 (9.99)	13.86 (11.40)	0.048
ΔFT (mean (SD))	21.12 (14.46)	22.53 (15.32)	19.67 (13.41)	0.242

Notes: 6MWD, six-minute walking distance; FIM, Functional Independence Measurement; FT, feeling thermometer; pts, points.

**Table 4:** Pre-post comparison between responders and non-responders according to 6MWD, FIM tot, FIM motoric, and FT.

	Overall			Responder			Non-Responder		
*n*	183			94			89		
	Pre	Post	*p*	Pre	Post	*p*	Pre	Post	*p*
6-MWD meter (mean (SD))	187.25 (133.77)	341.42 (131.80)	<0.001	250.35 (126.45)	426.10 (83.40)	<0.001	118.55 (104.45)	250.20 (111.41)	<0.001
FIM tot. (mean (SD))	98.12 (15.75)	113.51 (12.94)	<0.001	103.16 (12.33)	117.11 (8.30)	<0.001	92.93 (17.30)	109.51 (15.81)	<0.001
FIM motoric (mean (SD))	68.68 (12.89)	81.22 (10.75)	<0.001	73.04 (11.05)	83.72 (8.78)	<0.001	64.20 (13.20)	78.41 (12.08)	<0.001
FT degrees (mean (SD))	53.79 (16.99)	75.18 (13.14)	<0.001	52.96 (17.05)	75.24 (13.52)	<0.001	54.91 (16.87)	75.62 (12.18)	<0.001

Notes: 6MWD, six-minute walking distance; FIM, Functional Independence Measurement; FT, feeling thermometer.

**Table 5:** Correlations models excluding and including Forced Vital Capacity.

	Model without FVC (*n* = 181)		Model with FVC (*n* = 136)	
	Odds Ratios [95% CI]	*p*	Odds Ratios [95% CI]	*p*
ICU days	0.98 [0.92–1.04]	0.564	0.98 [0.91–1.06]	0.638
Hospital days	1.02 [0.96–1.07]	0.587	1.00 [0.93–1.07]	0.977
6MWD admission (per meter)	0.99 [0.99–1.00]	<0.001	0.99 [0.99–1.00]	0.002
Smoker (non-smoker)	0.57 [0.25–1.32]	0.193	0.64 [0.22–1.83]	0.403
CAD	1.50 [0.57–4.05]	0.415	2.39 [0.72–8.55]	0.163
Arterial fibrillation	2.76 [0.74–11.44]	0.140	3.85 [0.73–23.18]	0.121
FIM motoric (per each point)	0.96 [0.93–0.99]	0.020	0.93 [0.88–0.97]	0.004
Hemoglobin	1.01 [0.99–1.03]	0.474	1.01 [0.98–1.03]	0.633
Leukocytes	1.06 [0.99–1.13]	0.084	1.03 [0.93–1.13]	0.549
Potassium	1.57 [0.79–3.27]	0.206	1.32 [0.49–3.66]	0.578
FVC % pred			0.95 [0.93–0.97]	<0.001

Notes: ICU, Intensive Care Unit; 6MWD, six-minute walking distance; CAD, coronary artery disease; CIRS, Cumulative Illness Rating Scale; FIM, Functional Independence Measurement; FVC, forced vital capacity.

## Data Availability

Data supporting the reported results can be provided upon reasonable request by the corresponding author.
